# Organization Theory for Implementation Science (OTIS): reflections and recommendations

**DOI:** 10.3389/frhs.2024.1449253

**Published:** 2024-12-13

**Authors:** Sarah A. Birken, Jure Baloh, Michelle C. Kegler, Terry T.-K. Huang, Matthew Lee, Prajakta Adsul, Grace Ryan, Alexandra Peluso, Cheyenne Wagi, Aliza Randazzo, Megan A. Mullins, Kristin E. Morrill, Linda K. Ko

**Affiliations:** ^1^ Department of Implementation Science, Wake Forest University School of Medicine, Winston-Salem, NC, United States; ^2^ Department of Health Policy and Management, College of Public Health, University of Arkansas for Medical Sciences, Little Rock, AR, United States; ^3^ Emory Prevention Research Center, Rollins School of Public Health, Emory University, Atlanta, GA, United States; ^4^ Center for Systems and Community Design and NYU-CUNY Prevention Research Center, Graduate School of Public Health and Health Policy, City University of New York, New York, NY, United States; ^5^ Department of Population Health, New York University Grossman School of Medicine, New York, NY, United States; ^6^ Comprehensive Cancer Center, Department of Internal Medicine, University of New Mexico, Albuquerque, NM, United States; ^7^ Department of Population and Quantitative Health Sciences, University of Massachusetts Chan Medical School, Worcester, MA, United States; ^8^ O’Donnell School of Public Health, Simmons Comprehensive Cancer Center, University of Texas Southwestern Medical Center, Dallas, TX, United States; ^9^ LeCroy & Milligan Associates Inc., Tucson, AZ, United States; ^10^ Department of Health Systems and Population Health, School of Public Health, University of Washington, Seattle, WA, United States

**Keywords:** organization theory, implementation science, organization science, framework, healthcare

## Abstract

Organizations exert influence on the implementation of evidence-based practices and other innovations that are independent of the influence of organizations' individual constituents. Despite their influence, nuanced explanations of organizations' influence remain limited in implementation science. Organization theories are uniquely suited to offer insights and explain organizational influences on implementation. In this paper, we describe the efforts of the Cancer Prevention and Control Research Network's (CPCRN) Organization Theory for Implementation Science (OTIS) workgroup to equip implementation scientists with theory-guided understanding of organizational influences on implementation. We provide a set of recommendations for future efforts to enhance implementation through the use of organization theories and OTIS tools.

## Introduction

Evidence from diverse disciplines, ranging from sociology to applied mathematics, indicates organizations exhibit emergent properties that are distinct from those of the individuals who comprise organizations; organizations “display emergent behaviors that cannot be reduced to the intentions and values of individuals” (1). A vast literature exists on organizational identity and its influence, suggesting that organizations *themselves* (apart from any of the individuals who comprise them) are, in effect, key actors in implementation. Emerging applications suggest a demand for organization theory in implementation science (2), yet nuanced explanations of organizations' influence on implementation generally remain limited (3). Key questions often missing from implementation research and practice include, for example: What incentives and external pressures does an organization face? What turmoil has the organization experienced? Who comprises an organization's community, and is that community thriving? Understanding organizational influences on implementation requires characterizing the many dimensions of organizational identity (4). Comprehensive understanding of organizations must be broad in conceptualization, with an eye toward, for example, structure, processes, history, politics, and relationships with other organizations and the environments in which they operate.

Tropes such as “context is everything” and “if you've seen one Veterans Affairs (VA) [hospital], you've seen one VA” (5–15) ring true. It is also true that, comparing organizations may facilitate implementation. For example, the United States has an estimated 626 health systems (16). While health systems vary in terms of size, composition, financing, and location (16), they are similar in their rapid rate of expansion: Approximately 1,500 healthcare organizations were targeted for mergers and acquisitions between 2010 and 2019, and the number is increasing. Organization theory is uniquely suited to offer insights into optimal approaches to facilitating implementation in a rapidly changing healthcare landscape because organization theory.

“[I]s about the connections among phenomena…and delves into underlying processes…to understand the systematic reasons for a particular occurrence or [as is often the case in implementation] nonoccurrence…It often burrows deeply into neighboring concepts, or in an upward direction, tying itself to broader social phenomena…[A] good theory explains, predicts, and delights” (17).

*Contingency theory*, for example, suggests that optimal structure depends on the work an organization performs and features of the environment in which an organization operates. Thus, contingency theory implies that negotiation between a health system and acquired organizations will be critical in facilitating implementation efforts, as will addressing acquired organizations' unique needs (18). *Institutional theory* explains how environmental pressures from regulatory bodies, professional norms, and a propensity to adopt other organizations' approaches make organizations similar to one another. Understanding similarities across organizations allows us to address challenges such as reducing hospital readmissions (19). For example, hospital governance (e.g., leadership; quality committees) engage in efforts to reduce length of stay, yet surgeons as a group are compelled by professional standards and norms to discharge patients only when they are clinically ready. *Resource dependence theory* suggests that hospital governance's efforts might be more appropriately applied to establishing formal relationships with the multitude of external organizations into which patients are absorbed post-discharge (e.g., skilled nursing facilities, home health agencies, families) (20). When patients leave the hospital, their risk of readmission substantially increases because the discharging hospital becomes dependent upon these organizations, over which discharging hospitals have limited control. By engaging the organizations into which patients are absorbed post-discharge, hospitals can apply pressure on them to deliver care that will improve patient outcomes (21–24). Indeed, organization theory is instructive in conceptualizing the diverse community-based organizations with which healthcare organizations cooperate to optimize outcomes (e.g., food banks, home health agencies).

In this paper, we have two overarching aims: (1) to describe the efforts of the Cancer Prevention and Control Research Network's (CPCRN) Organization Theory for Implementation Science (OTIS) workgroup to equip implementation scientists and practitioners with an understanding of organizational influences on implementation (the OTIS framework) (25), and strategies to take into account organizational influences (OTIS abstraction forms) (3). (2) To provide a set of recommendations for future efforts to advance the use of organization theories and OTIS tools, and to enhance implementation efforts with organizations' powerful influence.

## OTIS workgroup activities and products

The OTIS workgroup was established to help advance the CPCRN mission. CPCRN was initiated in October 2002 with funding from the Centers for Disease Control and Prevention (CDC) and the National Cancer Institute (NCI) with a mission to (1) accelerate the adoption and implementation of evidence-based cancer prevention and control strategies in communities, (2) enhance large-scale efforts to reach underserved populations and reduce their burden of cancer, (3) deepen our understanding of the predictable processes that achieve those goals, and (4) develop the dissemination and implementation workforce in cancer prevention and control. The OTIS workgroup contributed to CPCRN's mission by establishing a goal of summarizing organization theories with relevance to implementation and developing and refining a framework comprised of organization theories' constructs. The OTIS workgroup's objective in pursuing these goals was to familiarize implementation scientists with organization theories and to facilitate organization theories' application to implementation science.

The OTIS workgroup was chartered in 2017 following a germinal, peer-reviewed debate paper in *Implementation Science* calling for the application of organization theory in the field (26). The OTIS workgroup has been comprised of a rotating roster of members with diverse content and methodological expertise from eight network centers and five affiliate institutions. Members collaborated on key activities including meetings at biannual CPCRN conferences, monthly workgroup calls, and *ad hoc* working meetings to pursue workgroup objectives (e.g., pairs of workgroup members reconciling abstractions from organization theory texts).

OTIS workgroup members collectively produced nine peer-reviewed articles and 18 peer-reviewed conference presentations; deliverables have included conceptual, methodological, and empirical work (Table 1). Following the publication of our germinal *Implementation Science* debate paper calling for the application of organization theory in the field, our work has focused on the three phases of OTIS framework development: (1) a survey of scholars with expertise at the intersection of organization and implementation science and abstraction of key organization theory concepts from organization theories, (2) development and refinement of the theoretical framework, and (3) applications of the abstraction forms and framework and the development of resources to facilitate OTIS framework use (e.g., interview guide; codebook; see Table 1). The three phases and OTIS outputs are summarized below.

**Table 1:** OTIS peer reviewed articles and conference presentations.

Phase 1: Identification and abstraction of organization theories
1. Birken SA, Bunger AC, Powell BJ, Turner K, Clary AS, Klaman SL, et al. Organizational theory for dissemination and implementation research. Implementation Science. 2017;12 (1):1–15. 2. Birken SA, Haines ER. Organization theory for implementation science. In: Rapport F, Clay-Williams R, Braithwaite J, editors. Implementation Science: The Key Concepts. London, UK: Routledge; 2022. p. 6. 3. Birken SA, Ko LK, Wangen M, Wagi CR, Bender M, Nilsen P, et al. Increasing access to organization theories for implementation science. Frontiers in Health Services. 2022;2:41. 4. Birken SA, Nilsen P. Implementation science as an organizational process. Health Care Management Review. 2018;43 (3):181. 5. Birken SA, Bunger AC, Powell BJ, Turner K, Slade A, Klaman S,…et al. The Case for Using Organizational Theory for Implementation Research. A poster presented at the 9th Annual Conference on the Science of Dissemination and Implementation. Washington, D.C. December 2016. 6. Birken SA, Bunger AC, Powell BJ, Turner K, Slade A, Klaman S,…et al. The Case for Using Organizational Theory for Implementation Research. A poster presented at the Organization Theory in Health Care conference. Berkeley, CA. May 2017. 7. Birken SA, Leeman J, Nilsen P, Bender B. Development of an Organization Theory Framework for Implementation. A poster presented at the Organization Theory in Health Care conference. Baltimore, MD. 2018 8. Birken SA. Development of a framework for organization theory for implementation science (OTIS). A poster presented at the Conference on the Science of Dissemination and Implementation. Washington, D.C. December 2018. 9. Birken SA, Leeman J, Charns M, Harrison MI. Organizational context in D&I: A rationale and guidance. A workshop at the Conference on the Science of Dissemination and Implementation. Washington, D.C. December 2018. 10. Nilsen P & Birken SA. Individual and collective determinants of implementation: Conceptualization and a framework for organizational theories for implementation science. An oral presentation at the Behavioural and Cognitive Theories for Knowledge Translation Course at University of Ottawa. Ottawa, Canada. June 2019. 11. Birken SA, Leeman J, Nilsen, P. Organizational Theory for Implementation Science: A rationale and guidance. A workshop at the European Implementation Conference. Rotterdam, Netherlands. October 2020. 12. Birken SA. Organizational Perspectives and Theories for Implementation Science. Implementation—Theory and Application in Practice Training. A course presented at Linköping University. Linköping, Sweden. October 2020. 13. Birken SA. Organizational Perspectives and Theories for Implementation Science. An oral presentation at the Implementation Science Affinity Group. Winston-Salem, NC. October 2022. 14. Birken SA. Improving care in dynamic systems: Lessons from organization theory. An oral presentation at the Knowledge Translation Canada National Seminar Series. A virtual meeting out of Canada. January 2023.
Phase 2: Development and refinement of OTIS framework
15. Birken SA, Wagi CR, Peluso AG, Kegler MC, Baloh J, Adsul P, et al. Toward a more comprehensive understanding of organizational influences on implementation: the organization theory for implementation science (OTIS) framework. Frontiers in Health Services. 2023;3. 16. Birken SA, Leeman J, Ko L, CPCRN members. Increasing access to organization theories for use in implementation science. An oral presentation at the Conference on the Science of Dissemination and Implementation in Health. Virtual Conference, December 2021. 17. Birken SA, Leeman J, Ko L, Peluso A, Wagi CR, Wangen M,…et al. Toward a more comprehensive understanding of organizational influences on implementation: The organization theory for implementation science (OTIS) framework. An oral presentation at the Dissemination and Implementation Conference. Washington, D.C. December 2022. 18. Baloh J, Adsul P, Arem H, Choy-Brown M, Fernandez ME, Huang TTK…et al. Organization Theory for Implementation Science (OTIS) Framework: Critical Review from Organization Scientists. A poster presentation at the Conference on the Science of Dissemination and Implementation in Health. Arlington, VA. December 2023 19. Birken SA, Nilsen P, Leeman J. Organization Theory for Implementation Science (OTIS): Advancing Implementation Science with A Framework of Organizational Theories. A workshop at the European Implementation Event. Virtual. February 2021. 20. 20. Birken SA. Increasing Access to Organization Theories for use in Implementation Science. An oral presentation at the Implementation Network Group at Linköping University. Linköping, Sweden. November 2021.

OTIS, organization theory for implementation science.

### Phase 1: identification and abstraction of organization theories

The goal of our conceptual work has been to advance the perspective that organizations have a critical yet underexplored influence on implementation, and poor access to organization theories has limited organization theories' contribution to the field of implementation science. Thus, our conceptual work introduced organization theories, their scope, past application, and potential application to implementation science. In our germinal paper, we demonstrated the benefits of applying organization theories to explain, facilitate, and evaluate implementation (26).

The OTIS workgroup published several papers reporting findings from our effort to identify organization theories with relevance to implementation science and abstract information from texts describing the identified organization theories (e.g., constructs; propositions). Our objective in publishing findings from organization theory identification and abstraction was to increase implementation scientists' access to largely underutilized organization theories. We published the abstraction forms on the CPCRN website (27) and described our process of identifying organization theories with relevance to implementation science through a survey of scholars with expertise at the intersection of organization and implementation sciences. We then described the subsequent process of abstracting information from the identified organization theories in a paper titled “Increasing access to organization theories for implementation science” (3). Briefly, abstraction forms provided an overview of the central tenets of organization theories, examples of applications of the organization theories to implementation science, constructs, propositions, potential relevance to implementation science (e.g., strategies), criticisms and/or bounds on the theory, and references to the articles from which data were abstracted. We then demonstrated the application of abstraction form content by applying propositions from three organization theories to practical examples in cancer prevention and control (e.g., using *transaction cost economics* to optimize fecal immunochemical testing through contracting with federally qualified health centers) (28).

### Phase 2: development and refinement of OTIS framework

Our objective in developing the framework was to facilitate the identification of factors at the intra- and inter-organization levels that are hypothesized to influence implementation but have seldom been considered in implementation science. We developed the framework by engaging scholars with expertise at the intersection of organization and implementation science in a virtual exercise in which they sorted 70 constructs from nine organization theories into domains (25, 29–32). Final domains included organizational characteristics (e.g., size, age); governance and operations (e.g., organizational and social subsystems); tasks and processes (e.g., technology cycles, excess capacity); knowledge and learning (e.g., tacit knowledge, sense making); characteristics of a population of organizations (e.g., isomorphism, selection pressure); and interorganizational relationships (e.g., dominance, interdependence).

OTIS workgroup members have initiated efforts to refine the OTIS framework. In May 2023, OTIS workgroup members conducted two focus groups to generate data to be used to refine the framework. In one focus group, at the CPCPRN spring meeting in Atlanta, GA, workgroup members LK and PA convened a meeting of members of the OTIS and Health Equity workgroups to identify opportunities to critique OTIS as it relates to equity. Reflections included (1) the importance of understanding deeply embedded racist histories and practices, which often vary across organizations, as a first step toward promoting equity; (2) the potential benefits of using a term other than racism (e.g., inclusivity) to allow for open conversation where the term is politicized; (3) the need for a follow-up meeting to discuss OTIS in detail because many of the participants required additional time to critique the framework; and (4) a recommendation to be strategic in identifying who should engage in the critique and via what process. Workgroup member SB conducted the second focus group at the 2023 Organization Theory in Health Care conference at Stanford University with a group of organization scientists. The 12 participants were asked to consider what should be added, removed, or revised in the framework. Workgroup member JB presented the findings at the 16th Annual Conference on the Science of Dissemination and Implementation in Health (33) (e.g., expand domains, edit constructs; see Table 2).

**Table 2:** OTIS feedback from organization scientist focus group (33).

Finding	Description
Expand domain	• Knowledge and Learning to reflect organizational adaptation, decision-making, and human resources (e.g., operation theory, knowledge management theory)
Edit constructs	• Organization-environment fit • Norms, standards, and guidelines • Team-building across organizations
Add domains	• Labor (role of labor, unions, and social movements; e.g., Marxism, high-performing work practice theories) • Competing Priorities (tradeoffs; e.g., resource mobilization theory) • Community Context (community engagement and decision-making)
Clarify overlap of constructs	• Across multiple OTIS domains and socioecological levels (inner and outer settings)
Improve usability for researchers and practitioners	• Providing operationalization guidance • Including examples • Allowing users to filter by construct/domain

OTIS, organization theory for implementation science.

### Phase 3: application and development of OTIS resources

OTIS workgroup members have also initiated efforts to operationalize the OTIS framework to further enhance its applicability in implementation science, deepening our understanding of how organizations influence adoption and implementation. Workgroup member KM received funding from the Consortium for Cancer Implementation Science public goods initiative focused on addressing key challenges in advancing the implementation science agenda in cancer and developing an interview guide and codebook based on the OTIS framework (34, 35). The detailed codebook contains descriptions of OTIS domains and constructs informed by research studies as examples of how they may be operationalized, both quantitatively and qualitatively. The moderator guide includes questions to elicit information about the potential influence of OTIS domains and constructs on implementation. Questions in the guide will be formulated for accessibility and relevance to non-academic individuals and can be adapted based on setting and priority population. The guide will be informed by 5–7 cognitive interviews with OTIS workgroup members. OTIS experts will review both tools to ensure they accurately and conceptually capture the 70 constructs represented in the framework.

Additionally, OTIS workgroup members have applied OTIS products to a variety of implementation research studies, including the application of 20 propositions from five classic organization theories—*complexity theory*, *contingency theory*, *institutional theory*, *resource dependence theory*, and *transaction cost economics*—to hypothesize relationships among outer setting factors, implementation strategies, and implementation outcomes in five case studies of evidenced-based tobacco control interventions (36). We also used organization theories to support the selection of strategies to target organization-level implementation barriers (e.g., fostering interactions among the multiple organizations throughout the community that serve patients; building a coalition to capture and share local knowledge, engage in local consensus discussions, and facilitate sense-making to develop and plan for implementation) (37). More recently, workgroup member SB applied OTIS to characterize the core functions (i.e., effectiveness-driving features) of evidence-based interventions with the goal of promoting their scale-up to new contexts and populations. For example, Wheeler et al. applied *transaction cost economics* to characterize an intervention with evidence of effectiveness in reducing cancer treatment-related financial toxicity as a compilation of governance structures that minimized the costs associated with financial navigation efforts (38). Wahlen et al. used *resource dependence theory* to explain how hospitals affiliated with a cancer program network improve quality by balancing the benefits of affiliation (e.g., shared resources) against the dependence that comes with affiliation (39) (e.g., shared decision-making processes). In a study led by workgroup member MM, investigators applied the OTIS framework to analyze data regarding the implementation of processes for documenting sexual orientation and gender identity in oncology programs (40).

## Discussion and recommendations for future work

Organization theories offer highly relevant yet largely untapped explanations of implementation. To increase access and application of organization theories in implementation science, CPCRN's OTIS workgroup collaborated over a seven-year period, producing multiple products. We began with the abstraction forms, which implementation scientists may use to familiarize themselves with organization theories identified by experts as relevant to implementation science. Ultimately, the group created a framework comprised of six domains representing 70 constructs across nine organization theories that implementation scientists may use to identify organization-level constructs hypothesized to influence implementation. OTIS workgroup members have begun to refine the framework to foster applications that advance health equity and adhere to concepts endorsed by organization scientists. Applications of OTIS products to date have enhanced diverse research studies at the intersection of cancer prevention and control and implementation science.

The OTIS workgroup has achieved its original goal of summarizing organization theories with relevance to implementation and developing and refining a framework comprised of organization theories' constructs. Yet, additional work is needed to advance OTIS's impact on theory, research, practice, and policy. Many scholars have called for increased attention to organization influences on implementation by highlighting relevant TMFs for assessing context (6, 41), offering taxonomies of features (42) and developing models for conceptualizing individuals' interactions with implementation context. Nevertheless, uptake of organization theories in implementation science continues to be limited, in part due to a lack of practical tools that implementation scientists can use to understand how and why organizational phenomena shape implementation. OTIS represents the first repository of such tools (e.g., abstraction forms; framework) of which we are aware.

Despite its potential contributions, some journals have been reluctant to publish new TMFs such as OTIS (43). Lacking viable alternatives for conceptualizing a more robust complement of organizational influences on implementation, we advocate for refining OTIS through application (25). Challenges to applying (and therefore refining) OTIS include the logistical and financial barriers to organizational research. Scholars have raised concerns for decades (44, 45), yet agencies (e.g., the National Cancer Institute) have only begun to grapple with the challenges of conducting organizational research (e.g., consenting and documenting enrollment data for research participants that are organizations rather than individuals) (46). To develop strategies that target organizational influences on implementation, research infrastructure and the funding environment must be adapted to accommodate organizational research, and the field must shift from a zero-sum perspective on TMFs to a more expansive approach to conceptualizing factors that influence implementation.

### Recommendations for theory

Work is needed to understand whether and how OTIS relates to the many extant theories, models, and frameworks (TMFs) in the field of implementation science. For example, the OTIS framework appears to have implications for Exploration, Preparation, Implementation, Sustainment (EPIS) framework, the Consolidated Framework for Implementation Research (CFIR), and the Practical, Robust Implementation and Sustainability Model (PRISM) (47), particularly in elaborating on their outer and inner setting domains; rigorous approaches are needed to systematically map OTIS domains and constructs onto those of EPIS, the CFIR and PRISM. Mapping OTIS onto extant TMFs could limit the proliferation of implementation TMFs while expanding extant TMFs' benefits (48). Such mapping would help to parse OTIS's unique contributions relative to extant TMFs and orient implementation scientists to OTIS using more familiar TMFs. For example, the OTIS framework presents an organization-level analogue to the primarily individual-level Theoretical Domains Framework (TDF), which synthesizes constructs from 33 psychological theories into 14 domains. Guidance for applying the TDF alongside OTIS would facilitate the multilevel implementation research for which scholars in the field have advocated (49). More broadly, further guidance about how to apply TMFs would enhance implementation scientists' ability to use the OTIS abstraction forms and the OTIS framework.

In addition to the potential to extend extant implementation TMFs, we intend for OTIS to encourage implementation scientists to more readily apply theory, as other scholars have called for (50). In contrast to models and frameworks, theories explain how and why constructs influence one another, identifying mechanisms underlying relationships. In turn, mechanisms suggest strategies that may be most impactful for improvement. Further, we intend for OTIS to enhance implementation scientists' consideration of collective influences on implementation. Despite experts' repeated calls for research on context, evidence regarding organizational drivers of implementation remains limited (51).

### Recommendations for research

We sought to facilitate the application of organization theory to guide the selection of implementation strategies that operate at the organizational level, in part to complement extant approaches' focus on implementation strategies that target individual-level change (52). As described above, we increasingly see OTIS's contributions to nuanced assessments of organizational influences in implementation research through empirical applications. In a new project led by workgroup member TH, OTIS will inform the study of implementation processes and outcomes in a diabetes intervention that integrates clinical and social care and digital health strategies. Efforts have also begun to develop resources to facilitate OTIS's application to qualitative implementation research, such as interview guides and codebooks. Additional efforts are needed to expand OTIS's application in quantitative work. For example, efforts are needed to identify or develop valid quantitative measures of OTIS constructs; such efforts would facilitate approaches to quantitatively testing relationships hypothesized by organization theories, conducting mixed-methods or quantitative formative evaluations, and testing implementation mechanisms. Additionally, empirical examples of how to apply the framework both prospectively (e.g., through the development of data collection instruments mirroring OTIS domains and constructs) and retrospectively (e.g., through a deductive application of OTIS to previously collected data) are needed to showcase different ways to utilize OTIS in line with research objectives. Another practical consideration for researchers is how to make findings from an application of OTIS actionable. For example, in a situation where key constructs affecting a program or intervention's implementation are identified, what does the next step of bridging findings to organizational or programmatic decision-making look like? What is the researcher's role in this process, what stakeholders should be included in these conversations, and what are the best ways to communicate learnings that considers organizational interest, readiness, and capacity? These questions will be important for researchers applying the framework in real-world settings to consider to maximize the utility of OTIS and cultivate trust with project partners through effective follow-through.

OTIS may be used to guide the selection and modeling of factors to be included in configurational comparative methods (CCMs) such as coincidence analysis (53). CCMs are premised on principles of conjunctivity (i.e., to bring about an outcome, several conditions must be jointly present); equifinality (i.e., multiple paths may exist to a given outcome); and sequentially (i.e., outcomes tend to produce further outcomes). Many organization theories' epistemological foundations are consistent with CCMs' principles because complex relationships are inherent to groups of individuals working toward common goals (54). For example, this approach could be used to examine community coalitions, which typically involve inter-organizational relationships across a broad range of community sectors (55). OTIS could guide selection of constructs to operationalize community context or to identify variables key to how organizations work together toward a common goal. CCM would help to identify which combinations of organization theory constructs are associated with coalition effectiveness.

Finally, OTIS may also inform systems science and policy-focused implementation research, including in community settings, allowing for more nuanced modeling of the factors and mechanisms in the outer and inner context (and their interplay), and helping to design policy- and systems-focused implementation strategies. For example, workgroup member TH received funding from the National Institutes of Health to apply systems modeling to understand how a human-centered design process can improve OTIS constructs to optimize community engagement, enhance neighborhood social environment, and ultimately improve community-level quality of life and mental wellbeing.

### Recommendations for practice

Practice settings may benefit from implementation strategies that organization theories indicate but are unaddressed by many extant implementation TMFs. OTIS could be used to support efforts to operationalize learning health systems (LHS), an increasingly popular yet poorly operationalized concept in U.S. healthcare (56). LHS are intended to optimize patient care through the integration of health system-generated data for improvement. Leveraging health system-generated data for improvement requires the technical, physical, relational, and cultural dimensions of organizing that organization theories help to conceptualize. CCMs may be particularly useful in identifying combinations of organization-level characteristics and activities that optimize care delivery in LHS. In addition, some organization theories include practical resources that healthcare professionals can use to operationalize organization-level strategies. For example, *network theory* scholars have developed a tool to assist practitioners in mapping and characterizing networks with external organizations (57). Approaches specifically designed to facilitate implementation practice should be amended to incorporate, explicitly organization theories. For example, Context-Driven Co-Design (CD2) identifies features of the implementation context that can be modified to facilitate implementation (58). The OTIS framework may aid CD2 users in identifying diverse organization-level factors not frequently captured in implementation TMFs.

Evaluators may also benefit from applying OTIS, for example, in the context of an implementation-focused evaluation. Consider the hypothetical evaluation of a statewide substance use program implemented across various health care settings. The OTIS framework may be useful as part of a pre-implementation evaluation to identify potential barriers and facilitators to effective implementation across sites considering external influences such as funding sources, economic conditions, public opinion and discourse shaping societal perceptions and expectations, and state and federal laws. The framework could also be applied after the program has already been implemented to identify potential risks to program fidelity across sites by providing a menu of potential factors that may affect implementation. As monitoring program fidelity and implementation is often a requirement for state and federally funded programs, evaluators may find it useful to have a starting place for such analyses, applying frameworks such as CFIR 2.0 (59) and OTIS to comprehensively assess both intra- and inter-organizational influences on implementation and fidelity and work with program staff to distill constructs to the most relevant and impactful determinants for continued monitoring and evaluation.

### Recommendations for policy

OTIS may support policymakers in their efforts to improve healthcare through regulation. In the context of cancer prevention and control, Centers for Medicare and Medicaid Services develops reimbursement policy that financially incentivizes specific types of care, and organizations such as the Commission on Cancer establish standards that compel affiliates to develop programs believed to optimize patient care. Healthcare organizations respond to multiple, often conflicting policies using approaches intended to minimize costs and optimize outcomes. Consequently, healthcare organizations often implement policies in ways that are unlikely to achieve policymakers' objectives, potentially contributing to unintended or harmful consequences (60). To improve their impact on care, policymakers would benefit from the understanding of organization-level factors that influence policy implementation that OTIS can provide. Thus, future work should tailor OTIS tools to policymakers' needs.

OTIS offers healthcare leaders tools for understanding the policy environment that they face and a set of strategies they can use to respond to the policy environment—either by knowledgeably navigating it or applying upward pressure to modify the policy environment. For example, OTIS could be used to orient users to the mimetic, normative, and coercive pressures (e.g., from accreditation bodies and federal, state and local government) in their organizational environment; describe the network of organizations with which it formally engages (e.g., health systems; professional organizations) and organizations it does not formally engage (e.g., local food banks; coalitions; advocacy groups). Future work is needed to translate OTIS into tools that healthcare leaders can use to understand their policy environment.

OTIS could also be used to identify opportunities for healthcare leaders to influence the policy environment to advance their goals. For example, organization theory conceptualizes organizations as capable of *learning* from policy to influence practice, and it conceptualizes organizations as capable of generating *knowledge* that can influence policy. Healthcare leaders can structure internal organizational features (e.g., governance; processes; technology) to apply learning from policy to practice, and they can generate practice-based evidence to influence policy (61). For example, Commission on Cancer's Cancer Liaison Physicians (CoC CLPs) in accredited hospitals could use their experience responding to CoC accreditation standards to engage with CoC leadership on potential refinements to CoC standards. OTIS's potential to inform policy implementation efforts will be ascertained through empirical application.

## Conclusion

To date, implementation scientists have lacked the resources needed to understand the unique influence of organizations on implementation. The CPCRN's OTIS workgroup developed products intended to promote the incorporation of organization theory into implementation science. In its seven-year history, the OTIS workgroup produced and refined summaries of organization theories with relevance to implementation science and a framework that synthesizes 70 constructs from nine organization theories into six domains. Despite mixed reception in the field and logistical and financial challenges associated with organizational research, OTIS products have been applied, and efforts continue to develop, refine, and operationalize the products. Future work, including broadening perspectives on OTIS's potential value-add and efforts to address the logistical and financial challenges associated with organizational research, is needed to promote OTIS's impact on implementation science in the realms of theory, research, practice, and policy.

## Data Availability

The datasets presented in this article are not readily available because data that led to the development of OTIS are available in previous publications and at reasonable request to the corresponding author. Requests to access the datasets should be directed to Sarah Birken, sbirken@wakehealth.edu.

## CPCRN OTIS Workgroup

University of North Carolina, Mary Wangen. University of North Carolina, Meghan O'Leary. Emory University, Michelle Kegler. Wake Forest University School of Medicine, Alexandra Peluso. University of New Mexico, Prajakta Adsul. New York University—CUNY, Terry Huang. University of Iowa, Grace Ryan. New York University—CUNY, Matthew Lee. Colorado School of Public Health, Linda Overholser. University of Minnesota, Mimi Choy Brown. University of California Irvine, Miriam Bender. Linköping University, Per Nilsen. University of Texas, Houston, Maria Fernandez. Wake Forest University School of Medicine, Cheyenne Wagi. Wake Forest University School of Medicine, Aliza Randazzo. University of Washington, Manal Masud. University of Arkansas for Medical Sciences, Jure Baloh. UT Southwestern, Megan Mullins. University of Arizona, Kristin Morrill. University of Washington, Barbara Baquero. University of North Carolina, Jennifer Leeman.
